# Traumatic dental injuries in adults attending a London-based trauma clinic in the UK: a seven-year survey

**DOI:** 10.1038/s41415-022-5313-4

**Published:** 2022-12-16

**Authors:** Serpil Djemal, Mohammadreza Aryafar, Aviva Petrie, Nectaria Polycarpou, Edward Brady, Sadia Niazi

**Affiliations:** 4141540551001 Consultant in Restorative Dentistry, King´s College NHS Trust, London, UK; 4141540551002 Consultant in Endodontics, King´s College NHS Trust, London, UK; 4141540551003 Honorary Associate Professor of Biostatistics, University College London Eastman Dental Institute, London, UK; 4141540551004 Clinical Lecturer and Honorary Consultant in Endodontics, King´s College London, UK

## Abstract

**Introduction** This survey reports the incidence of traumatic dental injuries in an adult population attending an adult dental trauma clinic in a London teaching hospital.

**Materials and methods ** Retrospective data were collected from patients attending an adult dental trauma clinic between 2012 and 2018.

**Results ** In total, 1,769 patients attended, with more men seen (1,030; 58.2%) compared to women (739; 41.8%) and this was statistically significant (p <0.05). The most common aetiological factor was an accidental fall (728; 41.15%), followed by assaults (413; 23.35%), bicycle accidents (253; 14.3%), sports injuries (132; 7.46%) and road traffic accidents (84; 4.75%). Lateral luxation (833) was the most common traumatic injury and this was followed by avulsions (362; 17%). Enamel-dentine fractures were the most common type of fracture injury (1,273; 64%).

**Discussion ** This retrospective survey attempts to report on the incidence of traumatic dental injuries in a London-based cohort of patients attending a specialised dental trauma clinic. In line with other reports, there were more men than women affected, which is probably attributed to behavioural activities.

**Conclusion(s)** Accidental falls are the most common cause of a traumatic dental injury, lateral luxation was the most common type of displacement injury and enamel-dentine fractures were the most common type of fracture injury.

## Key points

- Reports incidence of dental trauma in adults in the UK.
- Outlines the aetiology of dental trauma.
- Describes the differences between men and women in sustaining dental trauma.
- Lists the types and incidence of fracture injuries presenting to an adult dental trauma service.

## Introduction

Traumatic dental injuries (TDIs) are a significant public health problem. They also pose a significant socio-psychological impact on affected individuals and may affect their quality of life, with potential economic implications.^1^^,^^2^^,^^3^^,^^4^^,^^5^

The management of TDIs involves both short-term and long-term treatment considerations.^6^^,^^7^^,^^8^ What may appear to be a minor injury at the time of the traumatic incident may have significant long-term consequences. Such long-term complications may occur despite appropriate acute management and may not become apparent for many years. They include pulpal necrosis, root resorption and tooth loss in cases of severe trauma.^6^^,^^7^^,^^8^

The existing data on TDIs vary between countries, primarily due to differences in study design, the reporting of injuries and local environmental factors.^9^ The majority of studies on TDIs have been retrospective studies. A smaller number of prospective studies have been conducted. A major disadvantage of retrospective studies is that certain injuries could have been missed if signs and symptoms of trauma were not obvious at the initial clinical examination or at subsequent review appointments. They may also be dependent on the clinician's experience. A drawback of both prospective and retrospective studies is that injuries are not recorded if patients do not present for treatment. This is seen in the case of minor crown fractures.^5^

Most of the epidemiological studies report TDIs in children only^10^^,^^11^^,^^12^^,^^13^^,^^14^^,^^15^^,^^16^^,^^17^^,^^18^^,^^19^^,^^20^^,^^21^^,^^22^^,^^23^^,^^24^ and the data from these studies show a wide variation in the incidence of injury in the anterior dentition, ranging from 11.7%^12^ to 58.6%^20^ at the age of 12.

According to the International Association of Dental Traumatology, 33% of adults have experienced trauma to the permanent dentition.^25^ However, very few population-based studies of TDIs in adults have been undertaken. Furthermore, these studies vary extensively in the age range of the individuals who have sustained a TDI, as well as in the incidence.^26^^,^^27^^,^^28^ O'Mullane (1972) reported an epidemiology study in Cork, Ireland on a group of individuals aged 15-17 years and found that 19% had more than one traumatised permanent incisor.^29^ Holland *et al.* (1994) reported on a national survey of adult health in Ireland^30^ and found that the majority of adults aged 16-34 years had one tooth affected, with a higher incidence among men. Liew *et al.* (1986) looked at the number of patients attending with trauma to the anterior dentition in an out-of-hours clinic in Newcastle, Australia. They reported an occurrence of 24.9% in the 18-23 age group, with a male:female ratio of 2.6:1.^31^ In a national, USA, population-based study, Kaste *et al.* (1996) reported a incidence of 24.9% of incisor teeth injured among individuals aged 6-50 years. In their cohort, they found that half of the individuals had injured one incisor tooth. There was also a higher incidence in men compared to women and in older age groups.^27^ Locker *et al.* (2007) carried out a telephone survey in the Canadian province of Ontario and reported a 15.5% incidence of self-reported dental and oral injuries among adults aged 18-50 years. Again, there was a higher incidence in men, who also frequently experienced more than one injury.^32^ Clarkson *et al.* (1973), while investigating adults aged 15-60 years in London and Warrington (in the UK), found an incidence of 9.5% injured anterior teeth in 15-19-years-olds.^33^ From these studies, it is clear that there is a wide variation in incidence of traumatic dental injuries in the adult population.

Despite the potential seriousness and long-term sequelae associated with TDIs, there is a lack of prospective studies representative of the general population, especially in the UK. It is therefore beneficial to establish the frequency and causes of TDIs in a London-centric UK population in order to identify high-risk groups, plan for treatment demand and resources and to implement preventive strategies to try to reduce the economic and health burdens associated with dental trauma.

The aim of this study was to establish the overall incidence of TDIs affecting anterior teeth in an adult population attending a specialist clinic in the South of England. In addition, differences in incidence, sex, aetiology, type of teeth involved, types of injuries sustained and the pathway to access the specialist trauma clinic were investigated. The intention is to report on clinical outcomes of the patients in subsequent papers as part of Masters' research projects.

## Materials and methods

This retrospective survey used data collected from patients attending an adult dental trauma clinic (ADTC) at King's College Hospital between 2012 and 2018 (84 months). While King's College Hospital is a regional trauma unit in a large London teaching hospital, the patients included in this survey were walk-in patients with traumatic dental injuries, without any maxillofacial injuries.

The ADTC was serviced by a team of dedicated nurses and dentists ranging from consultants and specialists in restorative dentistry and/or endodontology (seven in total) to junior staff and postgraduate students. The majority of patients attending the ADTC were assessed and managed/treated by the consultants and specialists. Patients seen by the junior staff and postgraduate students were supervised by the same consultants and specialists. Diagnoses were made following clinical and radiographic assessment with two-dimensional radiographs using the dental trauma guide to inform all decisions.

Basic information was captured on a Microsoft Access database for identification purposes only. This included the mode of access to the clinic - whether patients attended the clinic as a ‘walk-in', without a referral letter, with a referral letter from a dental practitioner or from another hospital, or were directed to the clinic by their dentist, general medical practitioner or another hospital without a referral letter.

Information regarding patients' age, sex, date and time of the TDI, how it occurred and the teeth affected, along with any associated injuries sustained, were also noted on a data collection sheet, which was then transferred onto an access database. The data from this database were exported into a Microsoft Excel spreadsheet and anonymised before statistical analysis.

The data were analysed by Stata (StataCorp. 2021. Stata Statistical Software: Release 17. College Station, TX: StataCorp LLC). Bar charts were created in Microsoft Excel (Microsoft 365).

Chi-squared tests were carried out to compare relevant percentages and a significance level of 0.05 was used for all hypothesis tests.

## Results

Over the seven-year period, from 1 January 2012 to 31 December 2018, 1,769 patients were seen on the ADTC at King's College Hospital. All patients had at least one injury to their anterior teeth and the majority were from the Greater London area, with a few attending from further afield.

The age range of the individuals seen was 16-93 years and there were 1,030 men (58.2%) and 739 women (41.8%). The difference in incidence between men and women was found to be statistically significant (p <0.001).

**Fig. 1:**
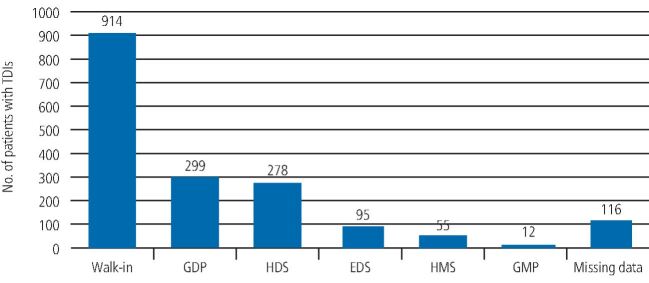
Graph showing the number of patients and referral source attending the ADTC with a traumatic dental injury (GDP = general dental practitioners; HDS = hospital dental services; EDS = emergency dental services; HMS = prison services; GMP = general medical practitioner)

The majority of the patients who attended the ADTC were ‘walk-in' patients (914; 51.7%), who attended without a referral letter. A further 299 patients (16.9%) were referred by their general dental practitioner, 278 (15.7%) were referred from another hospital, 95 (5.4%) from the emergency dental services, 55 (3.1%) from the prison dental services and 12 (0.7%) from general medical practitioners. There were 116 patients (15.3%) for whom the data were not captured, as seen in Figure 1.

**Fig. 2:**
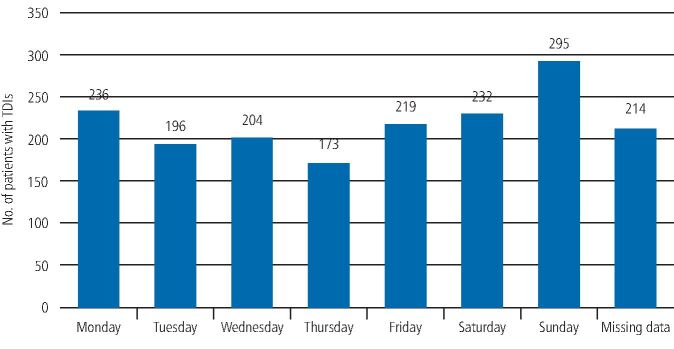
Graph showing the number of patients and the day of the week when the traumatic dental injury occurred

The day of the week that the TDI occurred is seen in Figure 2. The highest number of cases occurred on a Sunday, frequently in the early hours of Sunday morning.

**Fig. 3:**
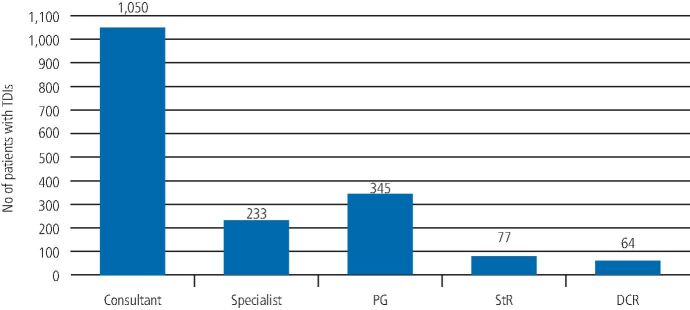
Graph showing the number of patients treated by the various clinical staff on the ADTC (PG = postgraduate student in endodontology; StR = speciality registrar in restorative dentistry; and DCT = dental core trainee in restorative dentistry)

At the ADTC at King's College Hospital, the majority of the acute management of the patients attending was provided by consultants (1,050; 59.4%) and specialists (233; 13.2%) in restorative dentistry and/or endodontology. Postgraduate students in endodontology also provided treatment under supervision (345; 19.5%), with some contribution from speciality registrars (77; 4.4%) and dental core trainees (64; 3.6%), as seen in Figure 3. This initial acute trauma management was carried out in the restorative clinic.

**Fig. 4:**
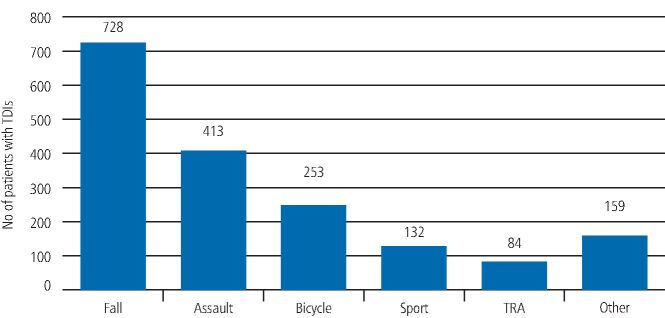
Graph showing the distribution of aetiology of all the traumatic dental injuries recorded from 2012-2018 (RTA = road traffic accident)

From this cohort of 1,769 patients, an accidental trip or fall was by far the most common cause of the traumatic dental injury and was documented as the aetiology in 728 cases (41.2%). This was followed by assault in 413 patients (23.4%), bicycle accidents in 253 patients (14.3%), sports injuries in 132 patients (7.5%) and road traffic accidents in 84 patients (4.8%). There were 159 patients (9.0%) for whom the aetiology of the TDI was not recorded (Fig. 4).

**Fig. 5:**
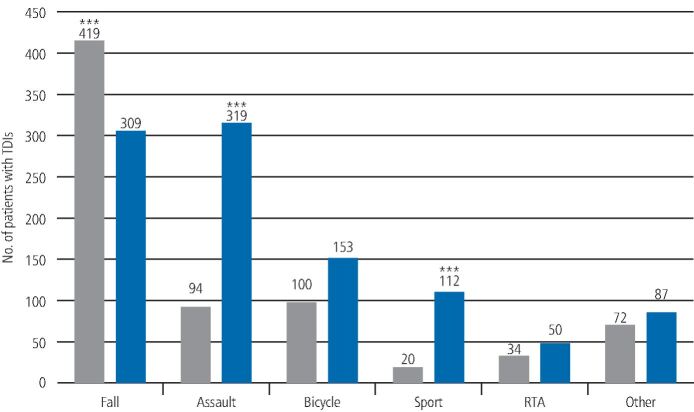
Graph showing the sex distribution of aetiology of all the traumatic dental injuries recorded from 2012-2018 (RTA = road traffic accident). There was a statistically significant difference (p <0.001) between men and women within the specific aetiology as indicated by ***

Further analysis of the data revealed highly significant associations between sex and aetiology (Fig. 5). There was a significantly greater (p <0.001) percentage of accidental falls in women (419; 57.0%) compared to men (309; 30.0%). There was also a significantly greater (p <0.001) percentage of assaults (319; 31.0%) and sports injuries (112; 11.0%) in men compared to women (94; 23.4% and 20; 3.0%, respectively). There was, however, no difference (p <0.05) between men and women in the number of bicycle accidents (153; 14.8% and 100; 13.5%, respectively) and road traffic accidents (50; 4.8% and 34; 4.6%, respectively).

In the 1,769 patients, there were 3,912 injured teeth and a total of 4,062 TDIs, as some teeth had sustained more than one injury.

**Fig. 6:**
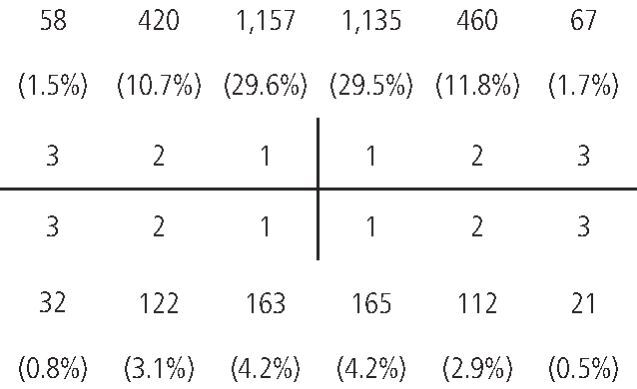
The distribution of the anterior teeth that sustained a traumatic dental injury expressed as a total and a percentage of the total number of teeth (1 = central incisor; 2 = lateral incisor; 3 = canine)

The teeth affected are seen in Figure 6 and, as expected, the maxillary anterior teeth were significantly more affected than the mandibular anterior teeth (p <0.001), although there was no significant difference between the left- and right-hand sides (p >0.05).

The upper central incisors were the most commonly injured teeth compared to the other anterior teeth (p <0.001), with 1,157 upper right central incisors (29.6%) and 1,135 upper left central incisors (29.0%) affected.

There were 420 upper right lateral incisors (10.7%) and 460 upper left lateral incisors (11.8%) that had sustained a TDI.

In the lower arch, both the right and left central incisors represented 4.2% (163 and 165, respectively) of the affected teeth, while the lower right and left lateral incisors accounted for 3.1% (122) and 2.9% (112) of the affected teeth, respectively.

Injuries to the canine teeth were significantly lower, with 1.7% (67) for the upper left canine, 1.5% (58) for the upper right canine, 0.8% (32) for the lower right canine and 0.5% (21) for the lower left canine (as tabulated in Figure 6).

**Fig. 7:**
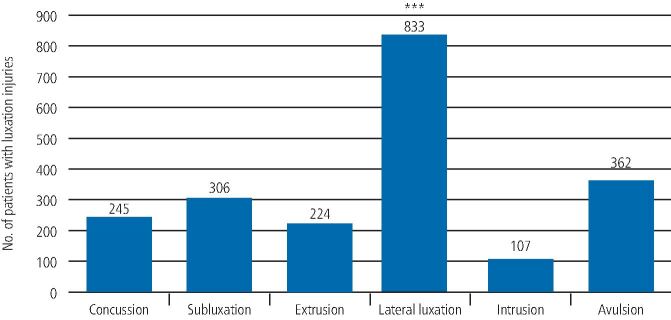
Graph showing the incidence of the luxation injuries. A statistically significant difference (p <0.001) is noted between lateral luxations and avulsions as indicated by ***

**Fig. 8:**
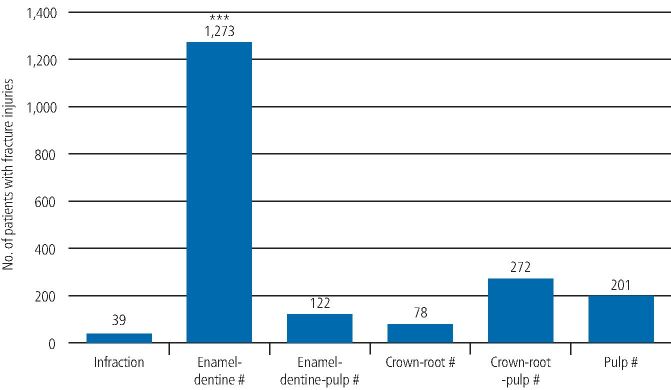
Graph showing the incidence of the fracture injuries. A statistically significant difference (p <0.001) is noted between enamel-dentine fractures and crown-root-pulp fractures as indicated by ***

Of the 4,062 TDIs, there were 2,077 luxation injuries (51.1%) and 1,985 fracture injuries (48.9%), as seen in Figures 7 and 8.

The incidence of the luxation injuries (2,077) as a percentage of the total injuries (4,062), as well as the incidence as a percentage of the luxation injuries only, can be seen in Table 1.

**Table 1:** Incidence of the different luxation injuries, shown as a percentage of the whole cohort, as well as a percentage of the luxation injuries only

**Luxation injuries**	**Number of teeth**	**% of teeth with a luxation injury as part of the total number of teeth injured (4,062)**	**% of teeth with a luxation injury as part of the luxation injuries only (2,077)**
Concussion	245	6	12
Subluxation	306	8	15
Extrusion	224	6	11
Lateral luxation	833	21	40
Intrusion	107	3	5
Avulsion	362	9	17
**Total**	**2,077**	**51**	**100**

From the luxation injuries, the most common type of displacement injury was lateral luxation (833), which comprised 40% of the total luxation injuries and was significantly greater than the next most common injury - avulsion (362) - which comprised 17% of the total luxation injuries (p <0.001).

Subluxation (306) was noted in 15%, concussion (245) in 12%, extrusion (224) in 11% and intrusion (107) in 5% of the total luxation injuries, as seen in Figure 7 and Table 1.

Lateral luxation was therefore the most prevalent traumatic dental injury and intrusion was the least common TDI.

The incidence of the fracture injuries (1,985) as a percentage of the total injuries (4,062), as well as the incidence as a percentage of the fracture injuries only, can be seen in Table 2. Enamel-dentine fractures (1,273) were significantly more common (p <0.001) than other types of fracture injury, constituting 64% of the fracture group. Enamel-dentine fractures were also significantly more common (p <0.001) than crown-root-pulp fractures (272; 14%) and were the second most common type of fracture injury seen. This was followed by root fractures (201; 10%), enamel-dentine-pulp fractures (122; 6%) and crown-root fractures (78; 4%). Infraction was the least common type of fracture injury, seen with a significantly lower incidence (2%) compared to the least common fracture - the crown-root fracture (p <0.001) - as seen in Figure 8 and Table 2.

**Table 2:** Incidence of the different fracture injuries, shown as a percentage of the whole cohort and as a percentage of the fracture injuries only

**Fracture injuries**	**Number of teeth**	**% of teeth with a fracture injury as part of the total number of teeth injured (4,062)**	**% of teeth with a fracture injury as part of the fracture injuries only (1,985)**
Infraction	39	1	2
Enamel-dentine	1,273	31	64
Enamel-dentine-pulp	122	3	6
Crown-root	78	2	4
Crown-root-pulp	272	7	14
Root	201	5	10
**Total**	**1,985**	**49**	**100**

## Discussion

This survey, which includes a total of 1,769 adults aged 16-93 years, is the first of its kind carried out on a large scale, to attempt to determine the incidence of TDIs in a London-based UK population. These patients were seen between 2012 and 2018 (84 months) at the ADTC at King's College Hospital in London, where patients can self-refer (‘walk-in') or be referred by their general dental practitioners, other hospitals with an emergency department or other services. The majority of the patients who attended the service were walk-in patients, demonstrating that the service is accessible and known about in the local health economy.

At the ADTC, a cohesive team of experts from the department of restorative dentistry and endodontics assessed and treated the patients. These included consultants, specialists, postgraduate students, speciality registrars and core dental trainees (junior staff).

Traumatic injuries to teeth, unlike other injuries in the body, are not self-healing and can have unpredictable prognoses. Timely diagnosis, appropriate treatment and follow-up are essential to minimise the social and economic impact of these injuries. The nature of damage is based on several factors, including the magnitude and direction of the force on impact and the shape of the injuring ‘object'. Also, certain predisposing factors may result in the patient having a higher risk of injury, for example, an overjet greater than 3 mm or incompetent lips.^9^^,^^34^^,^^35^^,^^36^^,^^37^

This study demonstrated a significantly higher occurrence of TDIs in men compared to women. This finding is consistent with other studies carried out either on adults or children, reporting a male: female ratio ranging from 1.5:1.0 to 2.5:1.0.^4^^,^^27^^,^^30^^,^^32^^,^^38^^,^^39^ This could be attributed to behavioural factors, such as greater propensity for men to be involved in fights, contact sports and road traffic accidents.^39^^,^^40^^,^^41^^,^^42^

The vast majority of studies have reported that the most frequent causes of TDIs are accidental falls, sports activities, bicycling and traffic accidents. Looking at the aetiology of the TDIs in the King's College Hospital group of patients, we also found that accidental falls were the most common cause of the TDIs seen and this is consistent with findings of other studies.^9^^,^^26^ Furthermore, accidental falls were more common in women compared to men.

The second most common aetiology in the King's College Hospital cohort of patients was assault, with a much higher incidence in men compared to women. This finding is in contrast to other studies, where physical violence was found to be the least common aetiology up to 6.6%,^9^ compared to 23.4% in the King's College Hospital cohort. This difference in the proportion of aetiology can be attributed to the age groups studied and social and cultural factors, which vary in different regions of the world.

The third most important aetiology was cycling accidents. In the UK and especially in London, there is an increasing trend for cycling. With increasing facilities, including designated cycling lanes, the public are encouraged to use bicycles as a means of travel within the city. Previously, cycling accidents were commonly reported in school-age children;^43^ however, due to the changing cultural trend, this was found to be the third most common cause of dental trauma in this survey.

TDIs due to sporting activities were also one of the aetiological factors with a higher occurrence in men compared to women. This is most likely due to the greater involvement of men in high-risk contact sports. It is also possibly because of women's higher adherence to the wearing of protective equipment, such as mouth guards or helmets with face protection, which certainly reduce the incidence of TDIs.

In this study, road traffic accidents were the least common cause of traumatic dental injuries. This is consistent with the findings of other studies.

Alcohol consumption at the time of injury could have been informative and interesting given the adult cohort of patients. This was asked of all patients and anecdotally, it was felt that patients were not always happy to answer the question or were not honest in their answer, as they felt that they may be refused treatment if they had been drinking. As a result, the authors did not include this variable in their analyses.

Maxillary central incisors were the most frequently traumatised teeth, followed by maxillary lateral incisors. This is consistent with the findings of other studies, which suggest that these teeth are at higher risk of sustaining a TDI.^5^^,^^9^^,^^10^^,^^27^^,^^29^^,^^31^^,^^44^^,^^45^^,^^46^^,^^47^^,^^48^^,^^49^This might also be associated in certain cases with predisposing factors, such as increased overjet and incompetent lips.^34^ General public awareness about wearing well-fitting mouth guards for contact sports and implementation of rules regarding the wear of mouth guards might dramatically reduce the incidence of anterior teeth injuries.

Lateral luxation was the most prevalent traumatic injury, accounting for 833 (40%) of all luxation injuries. This was followed by avulsion (362; 17%). These findings are similar to the results reported by Kaste *et al.*,^27^ who found that, at age 21-30 years, approximately 5% of trauma calls were due to teeth missing as a result of trauma, whereas the incidence increased to 25% at age 41-50 years. Locker,^32^ while carrying out a self-reported survey on dental and oral injuries of an adult population (18-50 years) living in Canadian province of Ontario, also found that one-quarter (25.4%) of that population reported avulsion and 6.5% reported a luxation injury.

Enamel-dentine fracture was the most common type of fracture injury (1,273; 64%), followed by crown-root-pulp fracture (272; 14%) and root fracture (201; 10%). Kaste *et al.*,^27^ using the National Institute of Dental Research modified index (National Health and Nutrition Examination Survey III) for classification of traumatic injuries to incisor teeth, reported enamel fractures as the most prevalent trauma (45.8%), followed by dentine fracture (17.0%) and fractures involving the pulp (4.0%). This difference in the distribution of the injuries could be attributed to different classification criteria. However, most of the studies have reported uncomplicated crown fractures without pulp involvement as the most common type of injury to permanent teeth,^10^^,^^34^^,^^36^^,^^50^^,^^51^^,^^52^^,^^53^^,^^54^^,^^55^^,^^56^^,^^57^^,^^58^^,^^59^ which is consistent with the findings of this study.

This survey was designed to report on incidence only. Treatment outcomes will be the subject of future publications.

## Conclusions

This study provides insight into the incidence of TDIs seen over a seven-year period at an ADTC at King's College Dental Hospital. During this period, 1,769 patients attended, presenting with 4,062 injured teeth.

In 728 (41%) of cases, the cause of injury was an accidental fall. This was followed by assaults (413; 23%), bicycle accidents (253; 14.3%), sports injuries (132; 7%) and road traffic accidents (84; 5%). In 159 patients (9%) the aetiology was not recorded.

There were similar proportions of luxation and fracture injuries (2,077; 51% and 1,985; 49%, respectively) and maxillary incisor teeth were the most commonly traumatised teeth.

The patients in this study will have comprised of a small fraction of the total TDIs suffered by patients throughout the UK over this seven-year period. The high incidence of TDIs recorded, however, highlights the need for improved access to education and treatment across the whole country. This includes the provision of centres equipped to manage dental trauma and ongoing education for general dental practitioners.

Additionally, the public would benefit from education on prevention, together with the advantages and types of sports mouth guards available and the emergency management of avulsions.

Further research is needed on the effectiveness of the management of TDIs, as well as the incidence of long-term sequelae that will be inevitable.
